# Transcriptome-based discovery of pathways and genes related to resistance against *Fusarium* head blight in wheat landrace Wangshuibai

**DOI:** 10.1186/1471-2164-14-197

**Published:** 2013-03-21

**Authors:** Jin Xiao, Xiahong Jin, Xinping Jia, Haiyan Wang, Aizhong Cao, Weiping Zhao, Haiyan Pei, Zhaokun Xue, Liqiang He, Qiguang Chen, Xiue Wang

**Affiliations:** 1 State Key Laboratory of Crop Genetics and Germplasm Enhancement, Cytogenetics Institute, Nanjing Agricultural University, Nanjing, 210095, China; 2 Institute of Agro-biotechnology, Jiangsu Academy of Agricultural Sciences, Nanjing, 210014, China; 3 School of Biology and Food Engineering, Chuzhou University, Chuzhou, 239000, People’s Republic of China

**Keywords:** *Fusarium* head blight, Wangshuibai, NAUH117, Resistance, Transcriptome, Digital gene expression

## Abstract

**Background:**

*Fusarium* head blight (FHB), caused mainly by *Fusarium graminearum (Fg)* Schwabe (teleomorph: *Gibberellazeae* Schwble), brings serious damage to wheat production. Chinese wheat landrace Wangshuibai is one of the most important resistance sources in the world. The knowledge of mechanism underlying its resistance to FHB is still limited.

**Results:**

To get an overview of transcriptome characteristics of Wangshuibai during infection by *Fg*, a high-throughput RNA sequencing based on next generation sequencing (NGS) technology (Illumina) were performed. Totally, 165,499 unigenes were generated and assigned to known protein databases including NCBI non-redundant protein database (nr) (82,721, 50.0%), Gene Ontology (GO) (38,184, 23.1%), Swiss-Prot (50,702, 30.6%), Clusters of orthologous groups (COG) (51,566, 31.2%) and the Kyoto Encyclopedia of Genes and Genomes (KEGG) (30,657, 18.5%), as determined by Blastx search. With another NGS based platform, a digital gene expression (DGE) system, gene expression in Wangshuibai and its FHB susceptible mutant NAUH117 was profiled and compared at two infection stages by inoculation of *Fg* at 24 and 48 hour, with the aim of identifying genes involved in FHB resistance.

**Conclusion:**

Pathogen-related proteins such as *PR5*, *PR14* and *ABC transporter* and JA signaling pathway were crucial for FHB resistance, especially that mediated by *Fhb1*. ET pathway and ROS/NO pathway were not activated in Wangshuibai and may be not pivotal in defense to FHB. Consistent with the fact that in NAUH117 there presented a chromosome fragment deletion, which led to its increased FHB susceptibility, in Wangshuibai, twenty out of eighty-nine genes showed changed expression patterns upon the infection of *Fg*. The up-regulation of eight of them was confirmed by qRT-PCR, revealing they may be candidate genes for *Fhb1* and need further functional analysis to confirm their roles in FHB resistance.

## Background

*Fusarium* head blight (FHB) caused mainly by *Fusarium graminearum (Fg)* Schwabe (teleomorph: *Gibberellazeae* Schwble) not only reduces grain yield and quality, but is also a major safety concern when human and animal consume *Fusarium*-contaminated wheat products [1]. Development and utilization of wheat varieties with FHB resistance is one of the main breeding objects, especially in the warm and humid wheat-growing regions, and has been recognized as one of the most economical, environmentally safe, and effective strategies for disease control [2]. Wheat cultivar Sumai 3 and wheat landrace Wangshuibai are the two most important sources for FHB resistance worldwide. Characterization of the mechanism underlying FHB resistance and the efforts for breeding new varieties with both high FHB resistance and good agronomic traits have been very difficult because resistance to FHB was a complex trait [3] and exhibited several types of resistance such as resistance to FHB initial infection (type I) and FHB symptom spread within a spike (type II) [4]. Molecular mapping of quantitative trait loci (QTLs) controlling FHB resistance has been extensively reported in the past ten years. However, map-based cloning of major QTLs including *Fhb1*, which has been mapped to the specific genome region of chromosome arm 3BS, has not been successful [5]. Defense-related genes were transformed to susceptible or moderate susceptible wheat varieties in the aim of obtaining transgenic plants with enhanced FHB resistance. These genes included *beta-1, 3-glucanase* (*PR2*), *chitinase* (*PR3*), *wheatwins* (*PR4*), *thaumatin-like protein* (*PR5*), *α-1-purothionin* and so on [6-11]. However, the obtained transgenic wheat lines only showed relatively low level of improvement of FHB resistance.

Transcriptome analysis of wheat spikes during infection by *Fusarium* pathogen will help us to understand the mechanism underlying FHB resistance and identify genes related to FHB resistance, and finally propose effective strategies to breed resistant variety for better control of FHB. In the recent years, the *Fusarium*-wheat interactions have been studied by differential gene expression. Using wheat microarrays, Li and Yen suggested jasmonate and ethylene signaling pathway may mediate FHB resistance in wheat [12]. Using a wheat cDNA microarray consisting of 5739 expressed sequence tags (ESTs), higher expression levels for three PR genes, *PR-2*, *-4* and -*5*, after *Fg* infection has been observed in Sumai 3 compared with two FHB susceptible near-isogenic lines [13]. Using wheat microarrays, 14 wheat gene transcripts showed accumulation differences between the resistant and susceptible alleles at *Fhb1*[14]. Combined proteomic and transcriptomic approach, the FHB resistance was found to be associated with coordinated and ordered expression of diverse defense signaling pathways that involves the signaling molecules SA, JA, ET, PA, Ca^2+^ and ROS as well as altered secondary metabolism [15]. Despite of these achievements of knowledge about the genes and pathways associated with FHB resistance in the above transcriptome research, there may be limitations to secure the identification of key genes related to wheat FHB resistance.

Wheat EST sequencing efforts provided genomics information and EST data, and allowed the creation of commercial wheat microarrays for high throughput gene expression profiling of different tissues or organs at a certain condition. The most currently used Affymetrix Wheat Genome Array contains 61,127 probe sets representing 55,052 transcripts from diverse wheat tissues or about 22,000 unigenes for all 42 chromosomes in the wheat genome, and key genes involved in FHB resistance may not be included. Thus, a comprehensive description of the genes expression of wheat spikes during infection is necessary for the discovery of candidate genes related to FHB resistance. Sequencing and characterizing of a cDNA library constructed from *Fg* infected wheat spikes did enrich the understanding of gene expression profiling. However, due to the high cost and time consuming of Sanger sequencing, the number of transcripts represented in the global gene expression assay remained limited [16]. Over the past several years, the next generation sequencing (NGS) technology has emerged as a cutting edge approach for high-throughput sequence determination, promptly improved the efficiency and speed of gene discovery, and dramatically reduced the time, labor, and cost [17-19]. With this technology, we can easily get a large number of sequence data which could represent the gene expression profile of the tissue at a particular condition.

Genetic background interference is another important factor for gene discovery based on differential gene expression analysis. Upon the infection, the differences of gene expression patterns between FHB resistant and susceptible lines were partially due to their genetic background differences rather than the differences for specific resistance locus, even for those studies using near-isogenic lines [14]. Pairwise expression comparison between resistant wildtypes and their susceptible mutants enables us to minimize the genetic background interference and hence is of great value for simplifying such complicated traits as FHB resistance by studying dissected individual QTL or single gene effects. Chinese wheat landrace Wangshuibai, which is originally from the severe epidemic areas of FHB disease of the Middle and Lower Region of the Yangtze River Area of China, has been recognized worldwide as an excellent FHB-resistant source. Similar to that in Sumai3, the major QTL on 3BS (namely *Fhb1*) harboring FHB resistance was also present in Wangshuibai [20]. The QTL has not been cloned and the resistance mechanism remains unclear. By fast neutron irradiation, we obtained a mutant (namely NAUH117) from Wangshuibai with increased FHB susceptibility, and the mutant had a 3BS chromosome fragment deletion including the *Fhb1* region [21]. Although it is difficult to clone the *Fhb1* by map-based cloning due to the deletion of a large chromosome fragment in the mutant, by comparing gene expression patterns between Wangshuibai and NAUH117 during *Fg* infection based on transcriptome analysis, it is feasible to uncover the mechanisms underlying FHB resistance, especially mediated by *Fhb1*.

By NGS of the Illumina technology, a huge number of distinct sequences (designated as unigenes) from the equally-mixed total RNA from Wangshuibai spikes at four infection stages (0, 12, 24 and 48 hai of *Fg*) were obtained. The unigenes were then assigned to putative functions, classifications or pathways based on sequence similarity analysis against the sequences in the public database resources. The assembled and annotated transcriptome sequences will provide a global view of gene expression of Wangshuibai spikes during infection by *Fusarium* and are the basis for candidate gene discovery. Based on our transcriptome data, a digital gene expression (DGE) system was used to compare the gene expression profiles of Wangshuibai and NAUH117 at two infection stages (24 and 48 hai). The comparison allowed us to reveal the molecular mechanism underlying the different resistance phenotypes of different genotypes, and may facilitate better understanding of resistance mechanisms of wheat in response to *Fusarium* infection, especially that conferred by the *Fhb1* locus. This is the first published study on global expression profiling of FHB-related genes in common wheat using NGS technology. Our results may aid the identification of pathways and genes associated with resistance to FHB in wheat.

## Results

### Transcriptome characterization of Wangshuibai spikes during infection with *Fusarium graminearum* by high-throughput RNA sequencing

To obtain the global gene expression profile of Wangshuibai spikes during infection by *Fg* RNA samples from spikes at three infection stages, 12, 24 and 48 hours after inoculation (hai), as well as the non-inoculated spikes were prepared, equally-mixed and then sequenced using the Illumina sequencing platform. After raw reads filtering and quality check, about 54 millions of 90 bp reads were obtained with 52.94% GC percentage (Additional file 1: Table S1). Transcriptome *de novo* assembly was carried out with short reads assembling program Trinity, resulting in 165,499 unigenes, with 62,442 unigenes have a length of more than 500 bp (see Additional file 2: Figure S1 for details).

For annotation, all the unigenes were aligned by Blastx to the NCBI non-redundant protein database (nr) and the Swiss-Prot protein database, using a cut-off E-value of 10^-5^. In the two databases, 82721 and 50702 unigenes (49.98% and 30.64% of all unigenes) returned the above cut-off BLAST results, respectively. The number of all unigenes with match hits in the two databases is 83027, and most of unigenes (50406 out of 50702 unigenes, 99.4%) with match hits in Swiss-Prot protein database can also be found in nr database. In both databases, the proportion of unigenes with match hits increased with the increase of the length of the assembled sequence. For sequences longer than 2,000 bp, the match efficiencies was 97.2% and 86.7%, whereas it decreased to 76.3% and 53.2% for sequences ranging from 500 to 1,000 bp, and to 38.8% and 20.3% for sequences less than 500 bp (Additional file 2: Figure S2A). Those unigenes with no match hit in the protein database might be due to their too short sequence length. As for annotation of species distribution, 67.6% and 15.5% had top matches (first hit) with sequences from rice (*Oryza sativa*), 9.6% and 1.8% from maize (*Zea mays*), 5.7% and 1.4% from wheat (*Triticum aestivum*), 3.2% and 1.2% from barley (*Hordeum vulgare*) and 10.5% and 39.2% from others, respectively (Additional file 2: Figure S2B).

**Figure 1:**
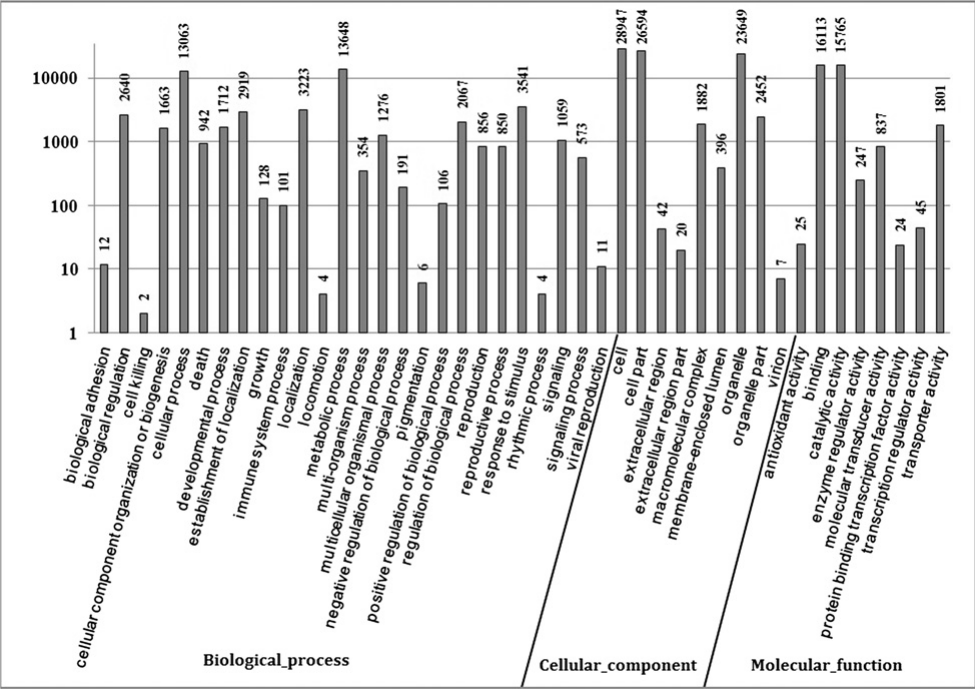
**Gene Ontology classification of unigenes.** 38,184 unigenes were categorized into 43 functional groups of three main categories: biological process, cellular component and molecular function. The y-axis indicates the number of genes in a category.

Gene ontology (GO) enrichment analysis was performed to classify the gene function of unigenes. Based on sequence homology, 38,184 sequences can be categorized into 43 functional groups consisting of three domains: biological process, cellular component and molecular function. In each of the three main categories of the GO classification, the cardinal terms are ‘Metabolic process’ (13648, 39.2%), ‘Cell’ (28947, 83.1%) and ‘Binding’ (16113, 46.2%), respectively. We also noticed a high percentage of genes from categories of ‘cellular process’ (13063, 37.5%), ‘cell part’ (26594, 76.3%), ‘organelle’ (23649, 67.8%) and ‘catalytic activity’ (15765, 46.2%) and only a few genes from terms of ‘Cell killing’(2, 0.01%), ‘locomotion’ (4, 0.01%), ‘pigmentation’(6, 0.02%), ‘rhythmic process ’(4, 0.01%) and ‘virion’ (7, 0.02%) (Figure 1).

**Figure 2:**
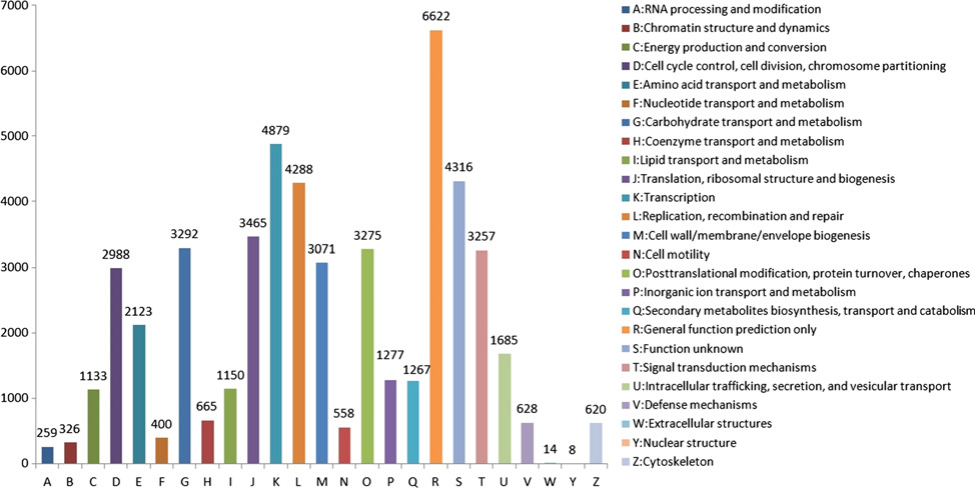
**Clusters of orthologous groups (COG) classification of unigenes.** 51,566 unigenes had a COG classification. The y-axis indicates the number of genes in a category.

For function annotation and classification of unigenes, we searched the annotated sequences for the genes involved in cluster of orthologous groups (COG) assignment, and 51,566 unigenes have a COG classification. Among the 25 COG categories, the cluster for ‘General function prediction’ represents the largest group (6622, 12.8%), followed by ‘Transcription’ (4879, 9.5%) and ‘Function unknown’ (4316, 8.4%). The categories of extracellular structures (14, 0.027%) and nuclear structure (8, 0.016%) represent the two smallest groups (Figure 2).

To characterize the complex biological behaviors for the transcriptome, KEGG (Kyoto Encyclopedia of Genes and Genomes) database was used analyze the pathway annotation of unigenes. In total, 30,657 sequences were assigned to 121 KEGG pathways (the top ten represented networks were listed in Additional file 1: Table S2). The pathways with most representation by the unigenes were Metabolic pathways (6965, 22.72%), Biosynthesis of secondary metabolites (3584, 11.69%) and Plant-pathogen interaction (3548, 11.57%). These annotations provided a valuable clue for investigating specific processes, especially those involved in plant-pathogen interaction.

### Digital gene expression (DGE) library sequencing and DGE tag annotation

Based on the Transcriptome sequence data, 6 DGE libraries were constructed to identify the gene expression profiles of Wangshuibai and NAUH117 spikes during *Fg* infection. The 6 DGE libraries include non-inoculated Wangshuibai and NAUH117 (designated as W0 and M0), Wangshuibai and NAUH117 at 24 hai (designated as W24 and M24) and 48 hai (designated as W48 and M48), respectively. Each library generated raw tags ranging from 5.90 to 6.18 millions. After removing the low quality reads, total tag numbers per library ranged from 5.62 to 5.90 millions and the number of tag entities with unique nucleotide sequences (distinct tags) ranged from 203,250 to 225,523 (Table 1). The saturation analysis was performed to check whether the number of detected genes keep increasing with the increase of sequence quantity (total tag number), it was found that when sequence quantity reaching 2 M or above, the number of detected genes almost ceased to increase (Additional file 2: Figure S3).

**Table 1:** **Statistics of DGE sequencing for 6 libraries from Wangshuibai (W) and NAUH117 (M) before inoculation (W0 and M0) and at 24 and 48 hour after inoculation (W24, W48 and M24, M48) of *Fg***

**Summary**			**W0**	**W24**	**W48**	**M0**	**M24**	**M48**
Raw tag	Total	number	6169913	6176043	5909902	5956701	6183284	5988697
	Distinct	number	506251	509167	484285	471505	484589	485332
clean tag	Total	number	5880158	5875394	5625082	5677385	5905722	5720549
	Distinct	number	225523	219763	210226	203250	217827	217240
Tag mapped to gene	Total	number	4029311	3855920	3816451	3770256	4092393	3929640
		% of tag	68.52%	65.63%	67.85%	66.41%	69.30%	68.69%
Tag mapped to gene	Distinct	number	106011	98948	94941	88609	101488	101264
		% of tag	47.01%	45.02%	45.16%	43.60%	46.59%	46.61%
Tags mapped to a unigene	Total	number	1569237	1509032	1482006	1439849	1598522	1521266
		% of tag	26.69%	25.68%	26.35%	25.36%	27.07%	26.59%
Tags mapped to a unigene	Distinct	number	24446	22422	22436	20858	24253	23682
		% of tag	10.84%	10.20%	10.67%	10.26%	11.13%	10.90%
Tag-mapped unigenes		number	24446	22422	22436	20858	24253	23682
		% of tag	14.77%	13.55%	13.56%	12.60%	14.65%	14.31%

To reveal the molecular events underlying the DGE profiles, the sequenced tags of the 6 DGE libraries were mapped to our transcriptome data containing 165,499 unigenes as mentioned above. Among these clean tags, 3.77–4.09 millions (66.41–68.52%) from the 6 libraries can be mapped to unigenes (Table 1). However, a proportion of tags which matched to more than one unigenes could be assigned to specific single unigene; they only represent the resulted all-inclusive expression levels of all unigenes with identical tag sequence. Those tags which could be mapped unambiguously to single unigenes indicated that the transcription data are reliable [22]. In this case, 20,858 to 24,446 distinct tags representing about a quarter of total tags (tags numbers from 1,439,849 to 1,598,522 or percentage from 25.36% to 27.07%) in each of 6 DGE libraries could be mapped unambiguously to one unigene in the reference database (Table 1). When considering all the libraries, 35,948 (21.72%) unigenes were detected in at least one of the libraries.

### Comparison of digital gene expression values with qRT-PCR analysis results

**Figure 3:**
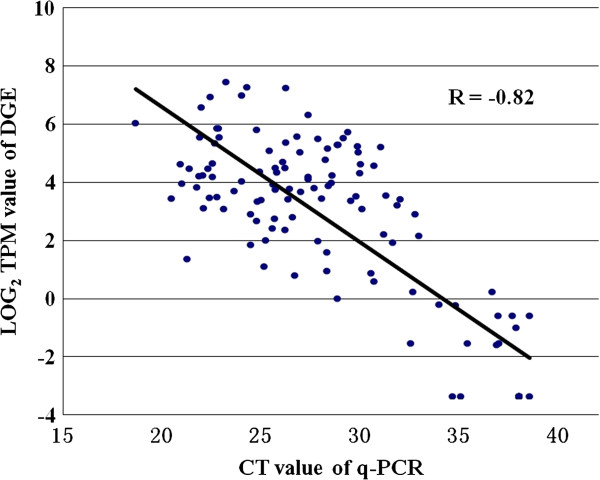
**Validation of DGE data by qRT-PCR.** Scatter plots indicate the Ct value of qRT-PCR analysis and the log_2_ TPM value of DGE for 144 data points from 24 genes in 6 samples. The Pearson correlation coefficient (R) was -0.82. The undetected tags in each library were plotted as 0.1 values.

Total sequenced tags from 6 libraries that matched to the 35,948 unigenes were classified as detected and reliable and designated as unambiguous clean tags. The gene expression level was determined by calculating the number of unambiguous tags for each gene and followed by normalization to the number of transcripts per million tags (TPM). Twenty-four unigenes with a range of expression values were selected for validation of gene expression levels estimated by TPM counts using quantitative RT-PCR (qRT-PCR) methods. The selected genes and their primers are listed in Additional file 1: Table S3. Expression levels of the 24 genes revealed by qRT-PCR analysis using 6 different RNA samples generated 144 data points. The Pearson correlation coefficient (R value) between the cycle threshold (Ct) value of the qRT-PCR and the log_2_TPM values of the DGE was -0.82, indicating that the gene expression levels by DGE analysis were positively correlated with those by qRT-PCR (lower Ct value refers to higher expression level), as was shown in Figure 3.

### Gene expression profile of Wangshuibai and NAUH117 during infection by *Fg*

**Figure 4:**
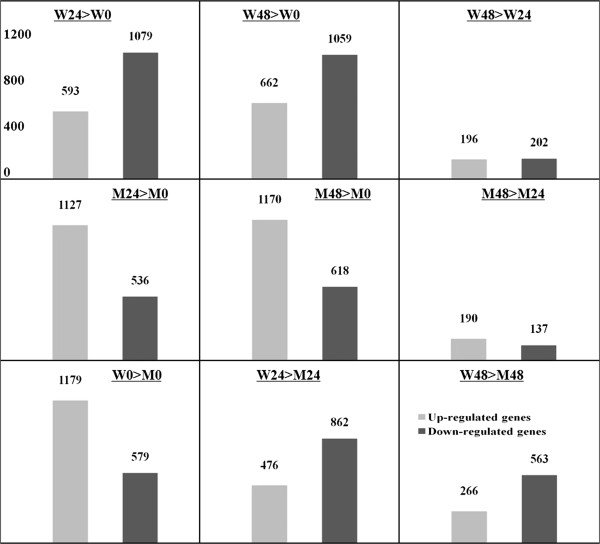
**Differentially expressed genes (DEGs) of each two libraries.** Differentially expressed genes were identified by filtering of the two-fold up- and down-regulated genes with FDR ≤ 10^-4^. Comparison of sample A with B was designated as A > B, in which A was the treatment and B was the control. W: Wangshuibai; M: NAUH117. 0/24/48 represents hours after inoculation. The y-axis indicates the number of DEGs for each comparison.

**Figure 5:**
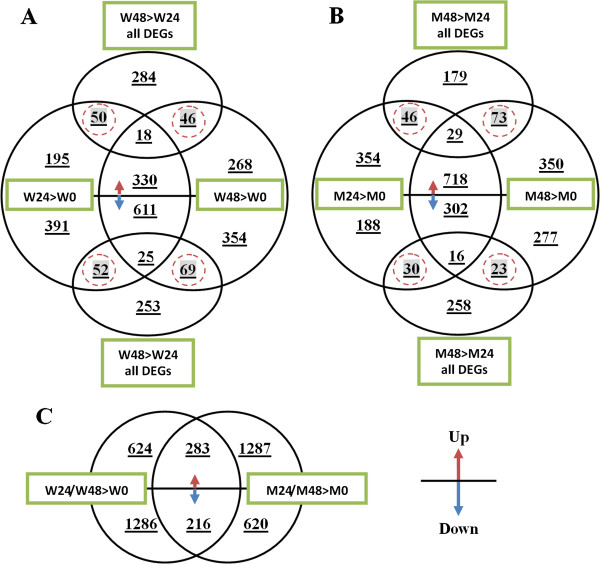
**Venn diagram comparison of differentially expressed genes (DEGs) at two infection stage (24 and 48 hour after inoculation) in Wangshuibai (A), NAUH117 (B) and between the two genotypes at both of two infections stages (C).** The meaning of “W”, “M”, “A > B” and 0/24/48 was the same as that described in Figure 4. The up or down arrows indicated that DEGs were up- or down-regulated. “all DEGs” meant all up and down regulated DEGs of the comparison. The specific genes for each genotype at the two infection stages were marked with red cycles.

The “FDR ≤ 10^-4^” and the absolute value of “log_2_ Ratio ≥ 1 or ≤ -1” was used as the threshold to identify and compare differentially expressed genes (DEGs) in and between Wangshuibai and NAUH117 at different infection stages. The generated 6 libraries were evaluated in 9 pairwise comparisons: W24 > W0, W48 > W0, W48 > W24, M24 > M0, M48 > M0, M48 > M24, W0 > M0, W24 > M24, W48 > M48 (When comparing sample A with B, designated as A > B, in which A is the treatment and B is the control. W represents Wangshuibai; M represents NAUH117; 0/24/48 represents hours after inoculation). Number of DEGs for each comparison was shown in Figure 4. Compared with their corresponding non-inoculated samples, thousands of genes were up- or down- regulated in the spikes of both Wangshuibai and NAUH117 at 24 and 48 hai. However, the number of the DEGs between the two infection stages for each genotype (W48 > W24 and M48 > M24) was much less (< 400 genes),showing that less changes for gene expression from 24 hai to 48 hai. Those DEGs only found at specific infection stage (marked with red cycles in Figure 5A and Figure 5B, respectively) may play special roles at certain infection stage.

In order to identify genes associated with FHB resistance, especially those related to *Fhb1,* FHB-responsive genes of Wangshuibai and NAUH117 upon the *Fusarium* infection were compared. We found that more genes were down-regulated and fewer genes were up-regulated in Wangshuibai than in NAUH117 (Figure 4). Only a small proportion of common DEGs were identified for the two genotypes, including 283 (12.9%) up-regulated and 216 (10.1%) down-regulated genes (Figure 5C). The great gene expression difference of the pattern between the two genotypes upon the infection was mainly due to their different FHB resistance level caused by the deletion at the *Fhb1* locus. Thus, those specific DEGs in Wangshuibai were assumed to be associated with FHB resistance mediated by or related to *Fhb1*.

### The difference of gene expression pattern between Wangshuibai and NAUH117 revealed by clustering analysis

**Figure 6:**
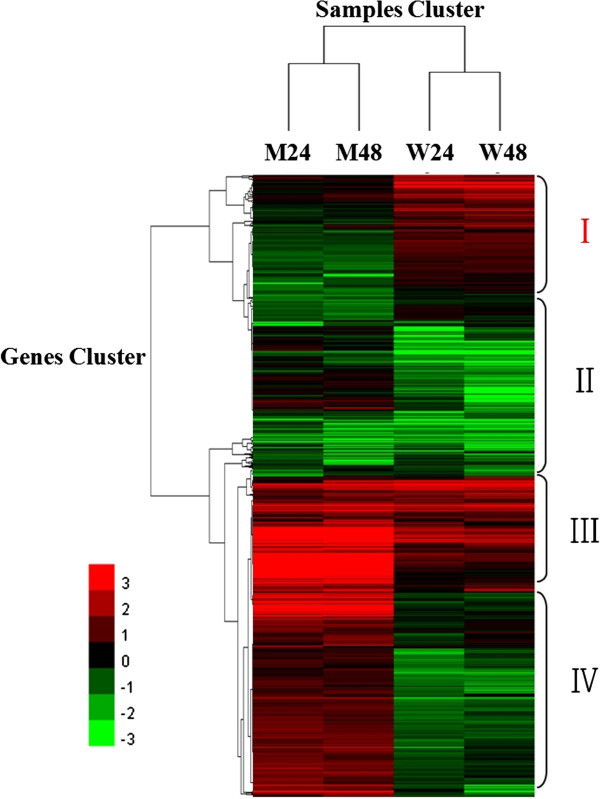
**Clustering analysis of gene expression patterns.** Heat map showed the gene expression clusters and samples clusters generated by the clustering affinity search technique (CAST) methods. Each line refers to data of one gene. The color bar represents the log_2_ of fold change values compared to mock, ranging from green (-3) to red (3).

All differentially expressed genes (FDR ≤ 10^-4^, fold change ≥ 2.0 or ≤ -2.0) in Wangshuibai and NAUH117 at 24 and 48 hai of *Fg* compared with non-inoculated samples were used to generate clusters using clustering affinity search technique (CAST). Samples clustering showed similar gene expression pattern at two different infection stages in the same genotype. Gene clustering showed that all DEGs can be clustered into three groups: Genes up- or down-regulated in both genotypes (II and III, Figure 6), which may be not critical for the FHB-pathogenesis responses mediated by *Fhb1*; Genes up-regulated in NAUH117 while down-regulated in Wangshuibai (IV, Figure 6); and genes up-regulated in Wangshuibai while down-regulated in NAUH117 (I, Figure 6). Those genes, only specifically up-regulated in Wangshuibai, may play important role in the FHB resistance that mediated by *Fhb1*.

### Differences of the molecular events in response to *Fg* infection in Wangshuibai and NAUH117

Previous studies have identified genes and pathways involved in or related to FHB resistance. In our data, we examined how these genes expressed in spikes of Wangshuibai and NAUH117 after infection of *Fg* toward the identification of genes or pathways critical for FHB resistance in Wangshuibai, especially that mediated by *Fhb1*.

### Pathogenesis-related proteins

**Figure 7:**
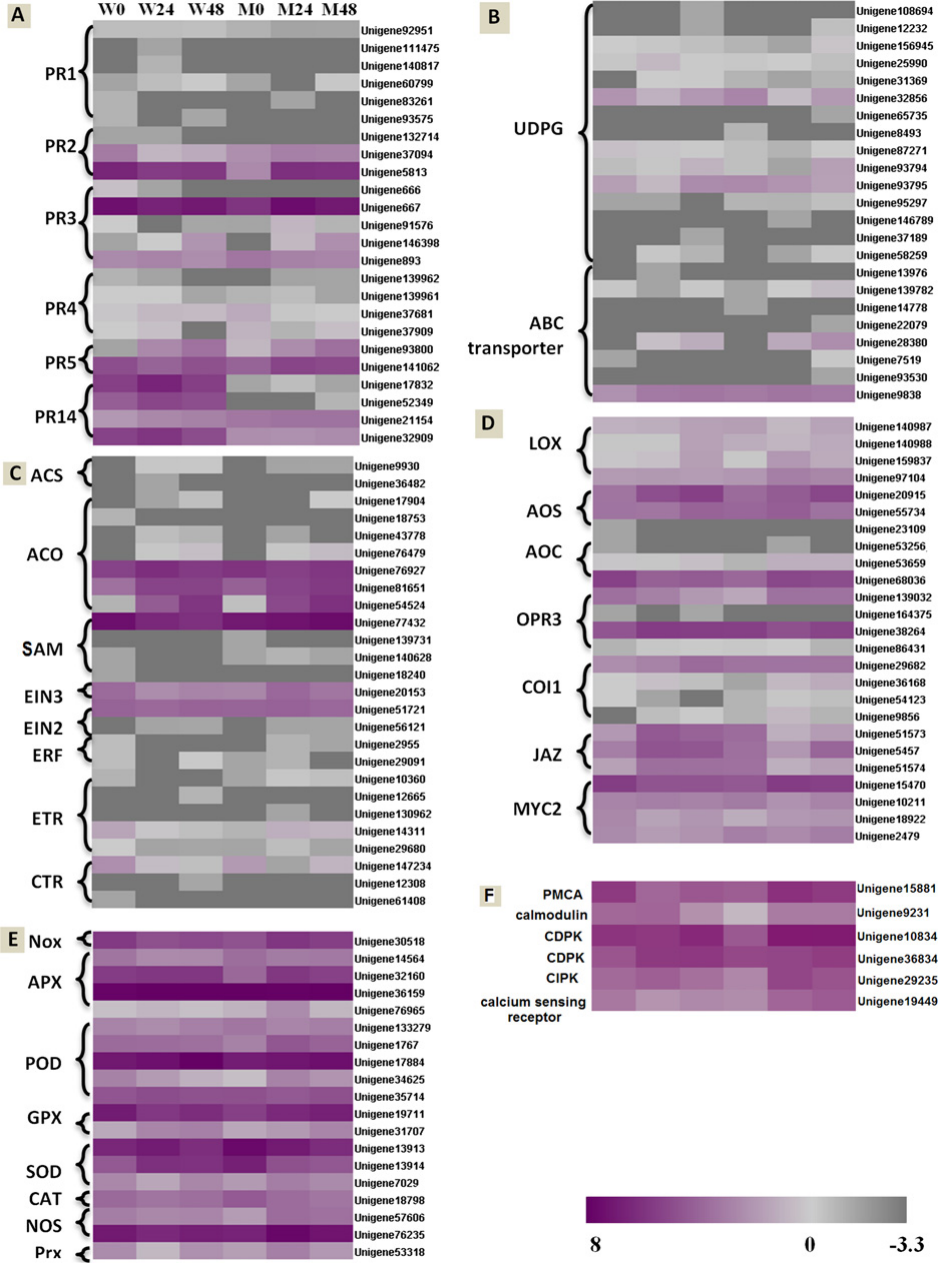
**Heat maps showed increased or decreased gene transcripts in specific classes in Wangshuibai (W) and NAUH117 (M) after *F. graminearum *infection, using corresponding non-inoculated samples as controls.** The bottom color bar represents the log2 of TPM values for each gene, ranging from gray (-3.3) to Purple red (8). The log2 of the undetected value, designated as 0.1 TPM, was -3.3 which the color was represented at the right end of color bar. Lighter color indicates more transcript accumulation. Comparisons of gene expression in Wangshuibai and NAUH117 after *Fg* infection for PR proteins (**A**), Detoxifying-related genes (**B**), genes involved in the ET pathway (**C**), JA pathway (**D**), ROS/NO pathway (**E**) and calcium signaling pathway (**F**).

The importance of pathogenesis-related proteins (PRs) in FHB resistance has been reported [10,11]. Among the 35,948 unigenes detected in at least one of the 6 DGE libraries, 24 unigenes encode PR proteins, in which there are 6 *pathogenesis-related protein 1.1* (*PR1*), 3 *β-1-3-glucanases* (*PR2*), 5 *chitinases* (*PR3*), 4 *vacuolar defense proteins* (*PR4*), 2 *thaumatin-like proteins* (*PR5*) and 4 *non-specific lipid transfer proteins* (*PR14*) (Figure 7A). Among the 3 *PR2* genes detected, 2 (unigene37094 and unigene5813) showed decreased transcripts accumulation in Wangshuibai and unigene5813 showed increased accumulation in NAUH117. One (unigene146398) out of the 5 *PR3* genes was induced in Wangshuibai and 2 (unigene667 and unigene146398) were induced in NAUH117. As for *PR5,* 1 (unigene93800) was up-regulated in Wangshuibai and 2 (unigene93800 and unigene141062) were up-regulated in NAUH117. Three (unigene17832, unigene52349 and unigene21154) out of the 4 *PR14* genes were induced in Wangshuibai while all PR14 genes remained unchanged in NAUH117. Moreover, compared with that in Wangshuibai, transcripts accumulation for these 3 genes at three infection stages in NAUH117 were much lower. No transcript change was detected for any of the *PR1* and *PR4* genes.

### Detoxifying-related proteins

Two classes of genes, the *UDP-glucosyltransferases* (*UDPG*) [23,24] and *ABC transporters*[25] were reported to detoxificate the *Fg*-produced trichothecenes, which act as virulence factor during the infection of *Fg* in wheat spikes [26]. In our research, 15 unigenes encoding *UDPGs* were detected with no transcript change after *Fg* infection except for unigene32856, which was repressed in both genotypes. Among the 8 unigenes encoding *ABC transporters*, unigene28380 was induced in both genotypes, while unigene9838 was induced in Wangshuibai but remained unchanged in NAUH117 (Figure 7B).

### Jasmonic acid-, ethylene- and salicylic acid- pathways

Plant defense in response to microbial attack is regulated through a complex network of signaling pathways that involve three signaling molecules: salicylic acid (SA), jasmonic acid (JA) and ethylene (ET) [27]. Conflict results have been reported for the effects of these pathways, either contributed or compromised the FHB resistance in wheat [12,15,28].

Twenty-five genes associated with the JA pathway were identified, including 14 genes responsible for JA biosynthesis [*lipoxygenase* (*LOX*), *allene oxide synthase* (*AOS*), *allene oxide cyclase* (*AOC*) and *12-oxo-phytodienoic acid reductase* (*OPR3*)], and 11 genes involved in JA signaling transduction [*CORONATINE INSENSITIVE1 (COI1*), *Jasmonate ZIM domain* (*JAZ*) and *MYC2*] (Figure 7C). One *LOX* gene (unigene159837), one *AOS* gene (unigene20915) exhibited increased accumulation in both genotypes. The *LOX* gene (unigene140988) and one *OPR3* gene (unigene38264) were induced only in Wangshuibai, and another *LOX* gene (unigene97104) and *OPR3* gene (unigene139032), and one *AOC* gene (unigene68036) were induced in NAUH117 while were repressed in Wangshuibai. This suggested that JA biosynthesis pathway was activated in response to the *Fg* infection in both genotypes. One *COI1* gene (unigene29682) and all the three *JAZ* genes (unigene5457, unigene51573 and unigene51574) were induced in Wangshuibai while their expressions remained unchanged or decreased in NAUH117. This suggested that the JA signal transduction pathway was normal in Wangshuibai but was blocked in NAUH117.

Twenty-six genes associated with the ET pathway were identified, including 12 genes responsible for ET biosynthesis [*S-adenosylmethionine decarboxylase* (*SAM*), *ACS synthesis* (*ACS*) and *ACC oxidase* (*ACO*)] and fourteen genes responsible for ET signaling [*ETHYLENE INSENSITIVE 2* (*EIN2*), *EIN3*, *ER-associated receptors* (*ETR*) and *ETHYLENE RESPONSE FACTOR1* (*ERF*)] (Figure 7D). Two *ACO* genes (unigene81651 and unigene54524) showed induced transcripts in both genotypes. One *SAM* gene (unigene77432) was repressed in Wangshuibai and remained unchanged in NAUH117. This suggested that ET biosynthesis pathway was activated in both Wangshuibai and NAUH117 after *Fg* infection. One *EIN3* gene (unigene20153) and one *ETR* gene (unigene14311) were up-regulated in NAUH117 but were repressed in Wangshuibai. *ChiB* gene (unigene667), the marker gene for ET pathway was also responsive to the *Fg* infection in NAUH117 and remained unchanged in Wangshuibai. The results showed that ET pathway was only activated in NAUH117 while not in Wangshuibai.

Ding et at (2011) reported that the SA pathway was involved in FHB resistance at early infection stage (within about 12 hai) [15]. In our research, none of the genes in the SA-mediated defense pathway, either for biosynthesis or signaling, was found to be differentially expressed both in Wangshuibai and NAUH117 (data not shown), indicating the SA pathway was not involved in defense response to *Fg* infection, especially at 24 or 48 hai.

### Reactive oxygen species and Nitric oxide

In plant defense response, reactive oxygen species (ROS) and Nitric oxide (NO) are thought to regulate programmed cell death (PCD) through the establishment of the hypersensitive reaction (HR) [29]. Sixteen genes related to the ROS production/scavenging systems were involved in the defense responses of wheat to *Fg* infection, including *NADPH oxidase* (*Nox*), *ascorbate peroxidase* (*APX*), *peroxidase* (*POD*), *glutathione peroxidase* (*GPX*), *superoxide dismutase* (*SOD*), *catalase* (*CAT*); Three genes related to the NO production/scavenging systems were involved in the defense responses, including *NO synthase* (*NOS*), *peroxiredoxin* (*Prx*) (Figure 7E). ROS-producing gene *Nox* (unigene30518) and NO-producing genes *NOS* (unigene57606 and unigene76235) were induced in NAUH117 while were repressed or remained unchanged in Wangshuibai. Consistently, many ROS- or NO- scavenging related genes were induced in NAUH117, including two *APX* genes (unigene32160 and unigene76965), two *POD* genes (unigene1767 and unigene34625), two *GPX* genes (unigene19711 and unigene31707), one *SOD* gene (unigene7029) and one *Prx* (unigene53318), while all ROS- or NO- scavenging related genes were repressed or remained stable in Wangshuibai except for one *GPX* gene (unigene31707) and one *SOD* gene (unigene13914) that were induced. The results suggested that ROS and NO were accumulated in NAUH117 while not in Wangshuibai.

### Calcium signaling pathway

**Figure 8:**
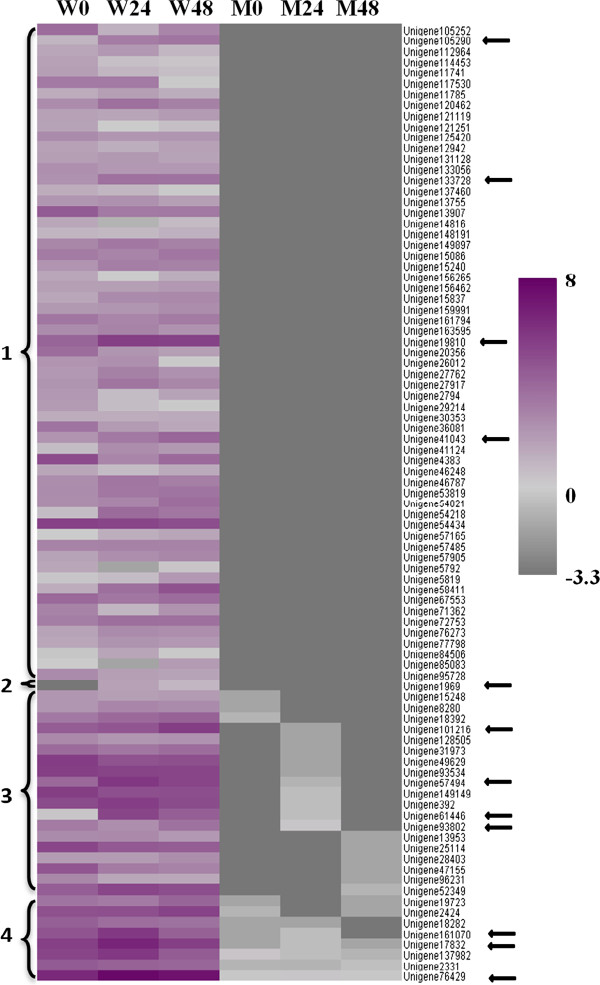
**The 89 genes with undetected expression in NAUH117 (M) compared to that in Wangshuibai (W) before and after the infection by *Fg*.** Each line refers to data of one gene. Each row refers to a cluster of genes for each library. The color bar on the right represents the log_2_ of TPM values for each gene, ranging from gray (-3.3) to Purple red (8). Lighter color indicates greater transcript accumulation from bottom to top. The log_2_ of the undetected value, designated as 0.1 TPM, was -3.3 which the color was represented at the bottom end of color bar. **1:** 61 unigenes can be detected in all 3 libraries of Wangshuibai that cannot be detected in NAUH117 in all of the 3 corresponding libraries; **2:** One unigene, which can be detected in W24 and W48 libraries rather than W0 library of Wangshuibai, cannot be detected in all 3 libraries of NAUH117; **3:** 19 unigenes can be detected in all 3 libraries of Wangshuibai but cannot be detected in at least two libraries of NAUH117; **4:** 8 unigenes, which were detected to have no more than 1.23 TPM in all three libraries of NAUH117, can be detected in all 3 libraries of Wangshuibai and exhibited a significantly higher gene expression level compared to that in NAUH117. The arrows indicated 12 unigenes which were up-regulated in Wangshuibai in response to the infection by *Fg*.

Accumulating evidence suggested that Ca^2+^ serves as a second messenger in host plant responses to pathogen [30]. Ca^2+^ ATPase (PMCA) is a transport protein in the plasma membrane of cells that serves as a regulator of the amount of Ca^2+^ within cells [31]. We found that a PMCA gene (unigene15881) showed contrary expression patterns in Wangshuibai and NAUH117 upon the *Fg* infection, i.e. up-regulated in NAUH117 while down-regulated in Wangshuibai (Figure 8F). The sensors of Ca^2+^ flux in plants in response to stresses, including a *calmodulin* (CaM, unigene15881), *calcium‐dependent protein kinases* (CDPKs, unigene10834 and unigene36834), a *CBL-interacting protein kinase* (CIPK, unigene29235) and a *calcium sensing receptor* (unigene19449), also showed different expression patterns in the two genotypes. They were all induced in NAUH117 and remained unchanged in expression in Wangshuibai except for one of *calcium‐dependent protein kinase* genes (unigene36834), which was induced in Wangshuibai while remained unchanged in NAUH117 (Figure 8F) . These implied the calcium signaling pathway may be not the key pathway for FHB resistance mediated by *Fhb1*.

### Identification of genes that were expressed in wildtype Wangshuibai while not in NAUH117

As mentioned above, a total of 35,948 unigenes were detected to be expressed in at least one of the 6 libraries. We found that 89 genes were expressed in at least two Wangshuibai libraries, but were not expressed or showed significantly decreased expression in at least two NAUH117’s libraries (Figure 8). We deduced that these differentially expressed genes in the two genotypes were due to the fragment deletion on the distal part of 3BS in NAUH117. Using Blastn search to the subject of wheat mapped ESTs database using a cut-off E-value of 10^-5^, these 89 unigenes were mapped to specific chromosomes (arms). Out of the 12 unigenes mapped to wheat mapped ESTs, 9 unigenes (75%) were mapped to chromosome arm 3BS (Additional file 1: Table S4), confirming the assumption that the undetected expression of these genes in NAUH117 was ascribed to its fragment deletion.

**Figure 9:**
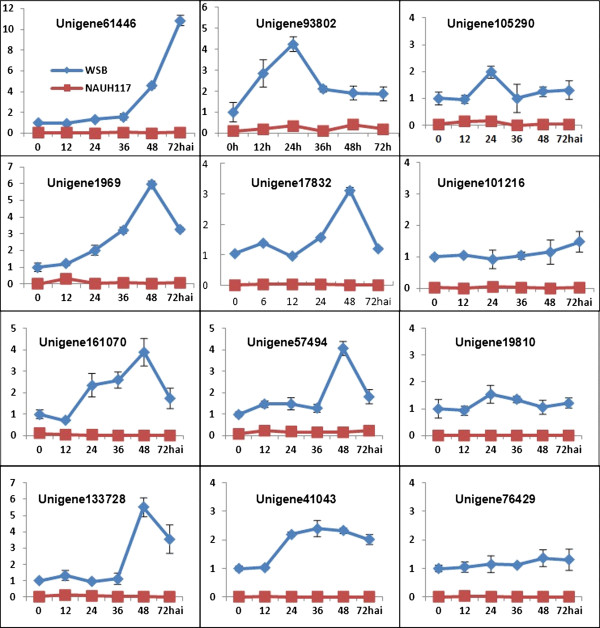
**Validation of DGE transcriptome results by qRT-PCR of the 12 genes which was up-regulated in Wangshuibai but unexpressed in NAUH117.** The expression levels on the y-axis were relative to the non-inoculated sample of Wangshuibai (0 h) after normalization with the wheat *Tubulin* gene. The experiment was repeated twice and data were presented as average ± S.D with n = 3; **hai:** hours after inoculation.

For *Fhb1* candidate genes discovery, 12 unigenes (indicated by arrows in Figure 8), which were up-regulated in Wangshuibai while not expressed in the mutant, were selected for qRT-PCR validation. We found that all the gene expression level in the mutant is very low compared with that in Wangshuibai. Eight out of 12 unigenes were verified to be induced in Wangshuibai during *Fg* infection and they may be related to *Fhb1* mediated FHB resistance and can be selected as *Fhb1* candidate genes for further study (Figure 9). These genes include a *receptor-like kinase*, a *lipid transfer protein*, a *NADP-dependent oxidoreductase*, a *glycine-rich protein*, an *ARK* protein, a *CBL-interacting protein kinase* and two genes with unknown function (Additional file 1: Table S5).

## Discussion

### Transcriptome of Wangshuibai spikes during *Fg* infection provided comprehensive knowledge for FHB resistance candidate gene discovery

Chinese Wheat landrace Wangshuibai has been recognized as an excellent FHB-resistant source worldwide. Recent studies have focused on the characterization of the mechanism of FHB resistance at molecular level [15]. Despite of the availability of wheat ESTs database from diverse tissues, a lack of available genetic and/or genomic information specifically for spikes of Wangshuibai during infection by *Fg* will be a barrier to further identify FHB resistance-related genes [16]. In this study, by Illumina sequencing, we obtained 165,499 unigenes from Wangshuibai spikes in response to *Fg* infection. About half of unigenes have Blastx hits in *nr* database, unigenes without annotation might be due to the relatively short length of their assembled sequences. However, 948 unigenes (9.1%) with sequence length longer than 1,000 bp have no match hits with any known sequence, they probably represented novel genes and will be supplemental genomic information for wheat. It should be pointed out that although a large number of potentially interesting genes were obtained from the transcriptome data, most of them were partial sequences of the corresponding genes and some unigenes were from different regions of the same gene. Due to short size or poor alignment, some sequences were even excluded from analysis, temperately. The availability of wheat genome sequence in the near future will offer an opportunity for transcriptome sequence assembly, and getting ‘real unigenes’. Anyway, our results provided an overview of gene expression profile of *Fusarium*-inoculated spikes of Wangshuibai and offer a valuable set of sequence data for further FHB resistant candidate gene discovery.

### Advantages and defects of DGE system combined with transcriptome in gene expression profiling study

DGE profiles of wheat spikes during *Fusarium* infection was the immediate direct application of our transcriptome data. The whole genome-wide gene expression analysis by DGE technique was based on computational analysis on 21-bp tags derived from the 3’ends of transcripts, an approach which has been proven successful in human and other species [32]. We used Wangshuibai transcriptome data as reference sequence to map the obtained DGE data for samples from both Wangshuibai and NAUH117. The transcriptome data from Wangshuibai is applicable for annotation of DGEs from NAUH117 because of their same genomic background except for the distal fragment deletion in 3BS of NAUH117 which leads to its FHB susceptibility [21]. We did not use wheat ESTs or unigenes in the public database as reference sequence for two reasons. Firstly, the public ESTs have a complicated genomics background, while our transcriptome sequences are specific for Wangshuibai or its mutant and make the DGE annotation more accurate. Secondly, many genes especially that are involved in FHB resistance may be not included in the public database due to the unsaturated genomic information. Our transcriptome sequence data offer an opportunity to improve the efficiency for DGE mapping.

The combination of transcriptome with DGE system has advantage over microarray technology, especially for the species without available genome sequences. The DGE, which generates digital rather than analog gene expression measurements, avoids many of the inherent limitations of microarray analysis. We believe microarray is still useful for accurate gene expression analysis at high throughput level [33], however, it is relative low sensitive for detection of rare transcripts and potentially can miss many targets that is not included on the array [34]. NGS-based transcriptome profiling is based on sequencing of relatively short reads, with extensive sequence data for new genes discovery and depth-of-coverage to detect and quantify even the rare transcripts [33,35,36].

This technology based on the relatively short sequenced reads (data form RNA-seq and Tag-seq) also had its drawbacks, especially for the organisms without reference genome sequence. The unigenes derived from de novo assembly of RNA-Seq data were partial sequences of the corresponding genes and gave rise to an inevitable bias for their annotation that more than two third (67.6%) of the blastx hits in nr database belonged to rice. As for Tag-seq data, the low mapping efficiency for DGE was the main problem that only a quarter of all DGE tags were mapped to the reference genes (Table 1). More thorough story could be gained when wheat genome is available.

### JA pathway played important role in FHB resistance of wheat and may be regulated by *Fhb1*

**Figure 10:**
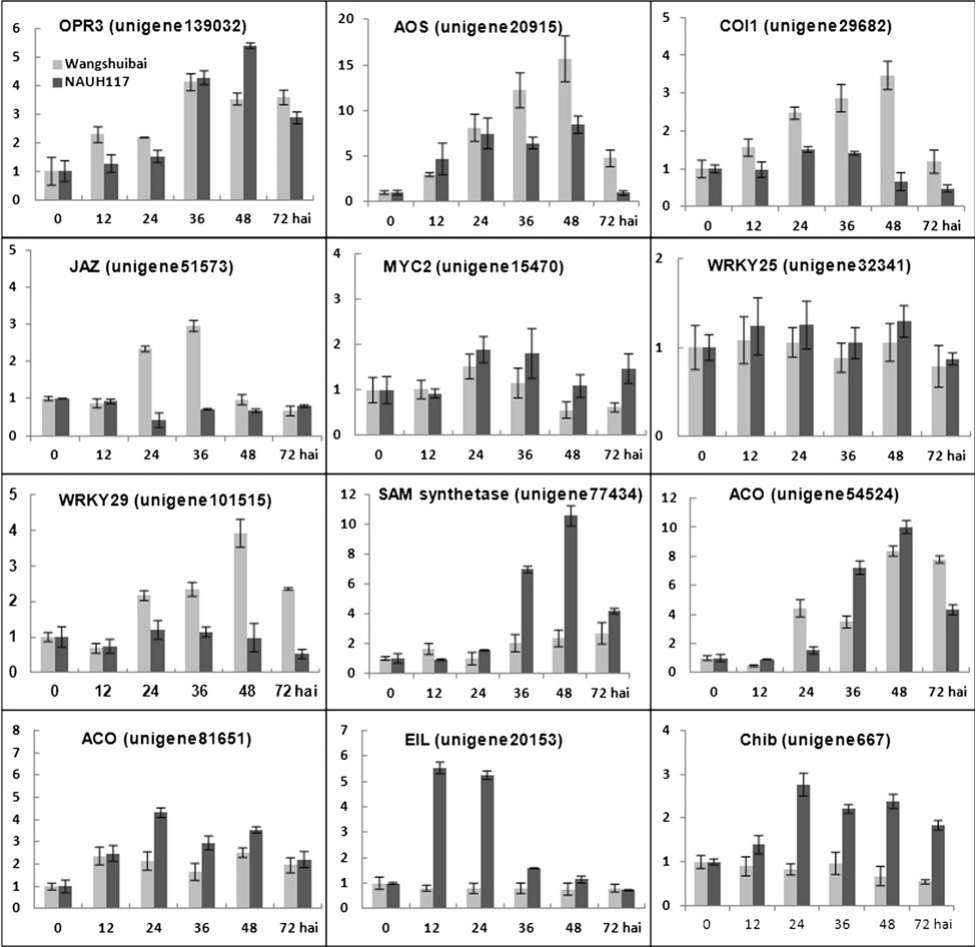
**Validation of DGE transcriptome results by qRT-PCR of the genes involved in the JA and ET pathways in Wangshuibai and NAUH117 after inoculation with *Fg*.** The expression levels on the y-axis were relative to non-inoculated sample from each genotype (0 h) after normalization with the wheat *Tubulin* gene. The experiment was repeated twice, and data were presented as average ± S.D with n = 3; **hai:** hours after inoculation.

**Figure 11:**
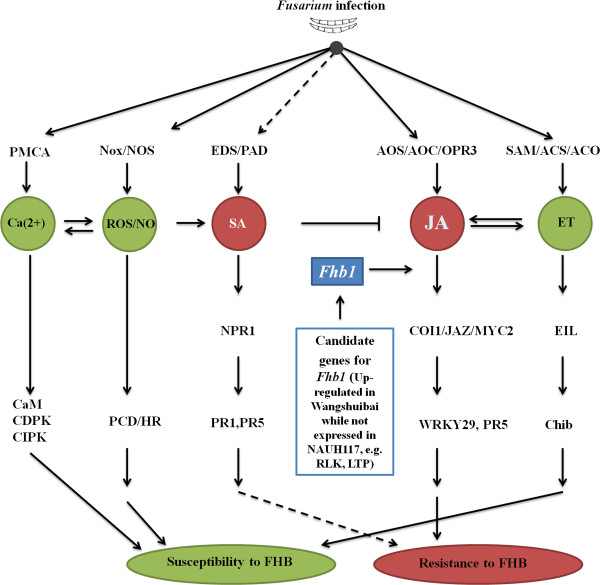
**A proposed working model of the SA, JA, ET, ROS/NO and calcium defense pathways in regulation of the resistance or susceptibility to FHB in wheat.** The dashed arrow indicates the potential roles SA pathway in response to the *Fg* infection at the early infection stage.

*Fg* infection exhibits a short biotrophic phase followed by the necrotrophic phase, as is a characteristic for hemibiotrophic pathogens [37]. Plant resistance to biotrophic pathogens is classically thought to be mediated by SA pathway, and by contrast, resistance to nectrotrophic pathogens is controlled by JA and ET pathways [38]. The positive role of JA pathway in FHB resistance of wheat has been reported [12,14,15]. Further study indicated that JA pathway may confer FHB resistance in the necrotrophic phase of pathogen [15]. Our results verified the importance of JA pathway in FHB resistance. JA pathway includes two steps, JAs biosynthesis followed by JA signal transduction, which leads to the activation of expression of JA-responsive genes [39]. From DGE data, JA biosynthesis pathway was activated in both Wangshuibai and NAUH117 and the result was confirmed by qRT-PCR for *AOS* (unigene20915) and *OPR3* (unigene139032) that were involved in JA biosynthesis (Figure 10). It was shown by the genes involved in JA signal transduction that JA signaling pathway was only activated in Wangshuibai while not in NAUH117. The result was confirmed by qRT-PCR analysis of gene expression patterns of *COI1* (unigene29682) and *JAZ* (unigene51573) (Figure 10). The unchanged expression of JA-responsive gene *WRKY29* (unigene101515) in NAUH117 after *Fg* infection confirmed the blocking of JA pathway and inactivation of JA responses in NAUH117 (Figure 10). *Non-specific lipid transfer protein* (*PR14*) is important in the transduction of lipid molecules, like JA [40]. Changed gene expression for *PR14* (unigene17832) only happened in Wangshuibai while not in NAUH117, and this might explain why the JA signaling pathway was blocked in NAUH117 (Figure 10). Compared to Wangshuibai, the deletion of *Fhb1* in NAUH117 may account for the blocking of its JA pathway, suggesting the possible regulation of *Fhb1* over the JA signaling pathway (Figure 11).

### ET pathway was not important for FHB resistance or even compromised the FHB resistance in wheat

Ethylene is a gaseous plant hormone that not only affected myriad developmental processes, but also led to fitness responses including programmed cell death [41]. The role of ET in host resistance appears to differ, depending upon the interaction system, improving resistance towards some pathogens, but increasing susceptibility towards others [30]. Similar to the JA pathway, the ET pathway also includes two parts, ET biosynthesis followed by ET signaling pathway [42]. Genes for both ET biosynthesis and signal transduction were induced in NAUH117 while not in Wangshuibai when infected by *Fg* and it was verified by qRT-PCR analysis for the ET pathway-related genes including *SAM* (unigene77432), *ACO* (unigene81651), *EIN3* (unigene20153) and *Chib* (unigene667) (Figure 10). It indicated that ET pathway was not important in the FHB resistance that mediated by *Fhb1*. The activation of ET pathways in NAUH117 may be a passive defense response to infection due to the lack of the most effective defense pathway. It was also reported that ET signaling compromised the resistance to *Fg* in *Arabidopsis*, wheat and barley through premature senescence. So, another speculation is that ET serves as an important passive regulator in the resistant lines. The inactivation of ET signaling pathways in Wangshuibai, which could decrease the number of cell death, may contribute the resistance at necrotrophic phase of *Fg*. However, Ding et al. [15] and Li and Yen [12] reported contrary results. They noted that *ACO*, the key gene in ET biosynthesis, showed different expression patterns between the resistant and susceptible genotypes, and was induced in the resistant genotype. In our research, one of the two *ACO* genes (unigene54524) was induced both in the resistant and susceptible genotypes. However, most of genes involved in ET pathway were responsive to the infection in susceptible rather than resistant genotype. Our results indicated ET pathway was not related to, or might even compromise the FHB resistance in wheat.

### ROS/NO and calcium signaling pathways were also not important in FHB resistance

Burst of NO and ROS was the main feature in plant defense against pathogen attack, in particular through the establishment of HR, which led to PCD to restrict the invading pathogen at the infection site [43,44]. Comparison of ROS and NO bursts in Wangshuibai and NAUH117 showed that ROS/NO production/scavenging systems were more active in NAUH117 than exhibited in Wangshuibai. We deduced ROS/NO pathway may play the similar role as ET pathway did in response to *Fg*. They contributed to the necrotrophic phase during *Fg* infection through PCD (Figure 11). Thus, the repression of NO and ROS bursts would lead to enhanced FHB resistance in wheat.

It was reported that there was relationship between ROS and calcium signaling in plant responses to stresses, that is, Ca^2+^ influx was important in the oxidative burst through activation of NADPH oxidase [45] and conversely, ROS regulated Ca^2+^ channels through the activity of NADPH oxidases [46]. Similar to the findings for ROS, calcium signaling pathway was responsive to *Fg* infection in NAUH117 while not activated in Wangshuibai. So, this pathway which was ready to leading to plant cell death was also repressed in Wangshuibai to keep its high FHB resistance level.

### Transcriptome-based discovery of candidate genes for or related to *Fhb1*

Transcriptome based on NGS generates absolute rather than relative gene expression measurements. Thus, our transcriptome data enabled the identification of unexpressed genes in NAUH117 compared to Wangshuibai, which was highlighted as one of the unique merits of the method. The undetected gene expressions in NAUH117 were related with the presence of the distal deletion on 3BS in NAUH117 [21]. These genes, especially 8 genes which were validated to be up-regulated in Wangshuibai upon *Fg* infection by qRT-PCR may be candidate genes for *Fhb1* or related to resistance mediated by *Fhb1*. A notable fact is that the annotation of 5 out of the 6 genes were defense-related, including the *receptor-like kinase* referred to R gene, the *lipid transfer protein* belonged to PR14 and PR2 respectively, the pathogen-related protein gene family, the *CBL-interacting protein kinase* was involved in calcium signaling, and a *glycine-rich protein* was the physical barrier to resist the pathogen infection. The *NADP-dependent oxidoreductase* was responsible for ROS burst, which may lead to susceptibility to FHB, thus was not important in our research. It was also worth to investigate the functions of the two genes without any annotated known function; they may represent the novelty of the molecular mechanism of FHB resistance in wheat. The cloning and functional analysis of part of these selected genes will be our next research focus for approaching the final goal of better understanding the mechanism of FHB resistance.

## Conclusion

Our transcriptome data provided comprehensive insight into gene expression profiles at two infection stages by *Fg* and facilitated the molecular mechanism study of FHB resistance in wheat. A putative network underlying resistance to FHB mediated mainly by *Fhb1* was proposed (Figure 11). Wangshuibai resists the spread of *Fg* in the infected spikelets mainly through the activation of the JA defense pathway, which was regulated by *Fhb1*. The ET, ROS/NO pathway and calcium signaling pathways, which were repressed by *Fhb1*, either were not involved in or were passive regulators of FHB resistance. SA pathway did not contribute to FHB resistance after the time point of 24 hai of *Fg*. Eight genes were identified to be *Fhb1* candidates and it would be of great value to further characterize their roles in FHB resistance. The cloning and functional analysis of two genes, a *receptor-like kinase* and a *lipid transfer protein gene*, was in progress in our lab.

## Methods

### Plant materials and fungal strain

Wangshuibai, a Chinese wheat landrace with high FHB resistance, was an important resistant source from southern China. NAUH117, a fast-neutron induced mutant from Wangshuibai, showed decreased typeII FHB resistance. Characterization of NAUH117 by genetic, molecular marker and cytogenetics analysis indicated the major FHB-resistance QTL *Fhb1* was deleted, and resulted in increased FHB susceptibility [21]. The materials were maintained by Cytogenetics Institute and grown in greenhouses at Jiangpu Experimental Station, Nanjing Agricultural University, Nanjing, Jiangsu Province, China.

The monosporic isolate of *F. graminearum* Fg0609 (kindly provided by Dr Xu Zhang, Jiangsu Academy of Agricultural Sciences) was used for fungal inoculations.

### Preparation of biological samples

Wheat spikes from Wangshuibai and NAUH117 were point-inoculated at anthesis with freshly prepared spore suspension. Each spike of 4 central spikelets was injected with 10 μl of conidial inoculant (10^5^ macro conidia per milliliter) and covered with a plastic bag to maintain humidity until sampling. Both non-inoculated spikes and inoculated spikes at 12, 24, 48, 72 and 96 hours after inoculation (hai) were sampled. Inoculations and sampling were conducted at 7 a.m. except for the sample at 12 hai at 7 p.m. A total of 12 samples (one treatment, two genotypes and six time points) were prepared for RNA extraction. Total RNA was isolated by using TRIzol Reagent (Invitrogen) according to the manufacturer’s protocol. RNA integrity was confirmed using the 2100 Bioanalyzer (Agilent Technologies) with a minimum RNA integrated number (RIN) value of 7.

### Preparation of cDNA library and Illumina sequencing for transcriptome analysis

The samples for transcriptome analysis were the mixture of equal amount of RNA from non-inoculated spikes and spikes at 12, 24 and 48 hai of Wangshuibai. Transcriptome library with fragments between 200 to 700 bp was prepared following the Illumina’s kits provided by manufacturer and sequenced on Illumina HiSeq™ 2000 using paired-end technology in a single run.

Prior to assembly, adapters and unknown or low quality bases which negatively affect bioinformatics analysis were discarded. Transcriptome *de novo* assembly was carried out with short reads assembling program Trinity [19], resulting in distinct sequence, designated as unigenes. The initial short reads data sets were available at the NCBI Short Read Archive (SRA) with the accession number: SRX212270. The assembled sequences (200 bp and above) have been deposited in the NCBI’s TSA database (TSA BioProject: 183717). The transcriptome shotgun assembly project has been deposited at DDBJ/EMBL/GenBank under the accession GAEF00000000. The version described in this paper was the first version, GAEF01000000.

The generated unigenes were analyzed by Blastx alignment search (e-value < 10^-5^) against protein databases nr, Swiss-Prot, KEGG and COG, and the best aligning results were used to determine sequence direction of unigenes. If results of different databases conflicted with each other, a priority order of nr, Swiss-Prot, KEGG and COG should be followed when deciding the sequence direction of unigenes. When a unigene happened to be unaligned to none of the above databases, software named ESTScan [47] was introduced to decide the sequence direction. For unigenes with sequence directions, we provided their sequences from 5’ end to 3’ end; for those without any direction we provided their sequences from assembly software.

Unigene sequences were aligned by blastx to protein databases nr, Swiss-Prot, KEGG and COG retrieved proteins with the highest sequence similarity for protein functional annotation and classification. For GO analysis of all the unigenes, we used Blast2GO [48] program to get GO annotation and WEGO [49] software for GO functional classification to understand the distribution of gene functions at the macro level.

### DGE library preparation and Illumina sequencing

Each sample of 6 μg of the total RNA was purified by Oligo (dT) magnetic beads adsorption. The mRNA was then used as template to synthesize the first and second-strand cDNA by Oligo (dT) primer. The 3’ ends of tags can be generated by two types of Endonuclease: *Nla*III or *Dpn*II. Usually, the bead-bound cDNA was subsequently digested with the restriction enzyme *Nla*III, which recognized and cut off the CATG sites. The fragments apart from the 3’ cDNA fragments connected to Oligo (dT) beads were washed away and the Illumina adaptor 1 is ligated to the sticky 5’ end of the digested bead-bound cDNA fragments. The junction of Illumina adaptor 1 and CATG site was the recognition site of *Mme*I, which is a type of Endonuclease with separated recognition sites and digestion sites. It cut at 17 bp downstream of the CATG site, and produced tags with adaptor 1. After removing the 3’ fragments with magnetic beads precipitation, Illumina adaptor 2 was ligated to the 3’ ends of tags, acquiring tags with different adaptors of both ends to form a tag library. After 15 cycles of linear PCR amplification, 105 bp fragments were purified by 6% TBE PAGE Gel electrophoresis. After denaturation, the single-chain molecules were fixed onto the Illumina Sequencing Chip (flowcell) for sequencing. Each tunnel generated millions of raw reads with sequencing length of 49 bp.

### Analysis and mapping of DGE tags

Raw sequences had 3’ adaptor fragments as well as a few low-quality sequences and several types of impurities. Raw sequences were transformed into clean 21 bp (CATG + 17 bp) tags by the following steps: (1) 3’ adaptor sequence was trimmed, resulting in 21 bp tags from 49 bp, of raw sequence (2) empty reads were removed (reads with only 3’ adaptor sequences but no tags); (3) low quality tags were removed (tags with ambiguous base calls); (4) tags of unusual length were removed, leaving only tags of 21 bp; (5) non-redundant tags were removed (each tag needs to be detected at least twice to be considered reliable). All clean tags were mapped to this reference database allowing no more than 1 bp mismatch. The number of mapped clean tags were calculated for each library and then normalized to TPM (number of transcripts per million clean tags).

### SYBR green real-time RT-PCR assay

All selected genes were conducted for SYBR Green Real-time RT-PCR assay. A real-time RT-PCR reaction (20 μl) included 20 ng cDNA, 0.2 μM of each prime, 1 × SYBR Premix ExTaq (TaKaRa Bio Inc., Japan). Reactions were performed on a Bio-Rad IQ single-color Real-Time PCR detection System (Bio-Rad, CA, USA) under the following conditions: 94°C for 30 s, 40 cycles of 94°C for 5 s, 60°C for 15 s and 72°C for 20s to calculate cycle threshold (Ct) values, followed by 95°C for 15 s, 60°C for 1 min, then 95°C for 15 s to obtain melt curves to ensure primer specificity. Pearson correlation coefficient was calculated between the cycle threshold (Ct) value of the qRT-PCR analysis and the log2 TPM values from the DGE analysis. The gene IDs and primer sequences were listed in Additional file 1: Table S3.

### Clustering analysis of differential gene expression pattern

Genes with similar expression patterns usually meant functional correlation. We performed cluster analysis of gene expression patterns with “cluster” software [50] and “Java Treeview” software [51] to construct the Hierarchical clustering (HCL) tree (using the Pearson correlation method with average linkage).

## Competing interests

The authors declare that they have no competing interests.

## Authors’ contributions

JX, XEW, AZC and HYW participated in the design of the experimental plan. WPZ, ZKX, HYP take part in statistical analysis. JX, XHJ, XPJ and LQH performed experiments. JX, XEW, XHJ and XPJ wrote the manuscript. All authors have read and approved the final manuscript.

## Supplementary Material

Additional file 1: Table S1 Output statistics of sequencing. Sample was the equally-mixed RNA from spikes at three infection stages, 12, 24, 48 hai of *Fg* and the non-inoculated spikes. **Table S2.** The network of unigenes in KEGG database. 30,657 unigenes were assigned to 121 KEGG pathways and the top ten representative networks were listed. **Table S3.** Information of primer sequences used in this study. **Table S4.***In silico* mapping of genes with undetected expression in NAUH117 compared with that in Wangshuibai. **Table S5.** Twelve unigenes were detected to be up-regulated in Wangshuibai while were not expressed in NAUH117 based on Digital Gene expression assay and 8 out of them were confirmed by qRT-PCR. Click here for file

Additional file 2: Figure S1 Length distribution of unigenes. **Figure S2.** The length (**A**) and species (**B**) distribution of unigenes and the proportion of unigenes with matches in nr and Swiss port databases. **Figure S3.** Saturation analysis of DGE sequencing. When the amount of tags reached 2M or higher, the increase of number of detected genes almost ceased for each library (indicated by the arrow). Click here for file
