# Supplementary material for: Transcriptome-based discovery of pathways and genes related to resistance against Fusarium head blight in wheat landrace Wangshuibai
Source: BMC Genomics. 2013 Mar 21;14:197. doi: 10.1186/1471-2164-14-197 (PMC3616903; doi:10.1186/1471-2164-14-197)
Supplement: Additional file 1: Table S1 — Output statistics of sequencing. Sample was the equally-mixed RNA from spikes at three infection stages, 12, 24, 48 hai of Fg and the non-inoculated spikes. Table S2. The network of unigenes in KEGG database. 30,657 unigenes were assigned to 121 KEGG pathways and the top ten representative networks were listed. Table S3. Information of primer sequences used in this study. Table S4.In silico mapping of genes with undetected expression in NAUH117 compared with that in Wangshuibai. Table S5. Twelve unigenes were detected to be up-regulated in Wangshuibai while were not expressed in NAUH117 based on Digital Gene expression assay and 8 out of them were confirmed by qRT-PCR. [file 1471-2164-14-197-S1.pdf]

Table S1. Output statistics of sequencing.

Sample was the equally-mixed RNA from spikes of Wangshuibai at three infection stages, 12, 24, 48 hai of *Fg* and the non-inoculated spikes.

Sample	Total Reads	Total Nucleotides (nt)	Q20 percentage	N percentage	GC percentage
Wangshuibai	54,117,052	4,870,534,680	94.82%	0.00%	52.94%

Table S2. The network of unigenes in KEGG database.

30,657 unigenes were assigned to 121 KEGG pathways and the top ten representative networks were listed.

	**Pathway**	**Unigenes with pathway annotation (Total: 30657)**	**Pathway ID**
**1**	Metabolic pathways	6965 (22.72%)	ko01100
**2**	Biosynthesis of secondary metabolites	3584 (11.69%)	ko01110
**3**	Plant-pathogen interaction	3548 (11.57%)	ko04626
**4**	Spliceosome	1649 (5.38%)	ko03040
**5**	Purine metabolism	994 (3.24%)	ko00230
**6**	Starch and sucrose metabolism	902 (2.94%)	ko00500
**7**	Protein processing in endoplasmic reticulum	895 (2.92%)	ko04141
**8**	Pyrimidine metabolism	874 (2.85%)	ko00240
**9**	Phenylpropanoid biosynthesis	788 (2.57%)	ko00940
**10**	RNA polymerase	645 (2.1%)	ko03020

Table S3. Information of primer sequences used in this study.

Gene IDs	Forwards primers	Reverse primers
unigene139032	GGAACCACGGATGGTGAATA	GGCAAGTCTGGATTGGACAG
unigene20915	TCTTGTGCCCTTGTGTGTGT	ATGGTGAGTGGCTGTGAATG
unigene29682	CCTTTGGCAAGAACCGTATC	ATCAAAGCACGGAGCAACTT
unigene51573	CCGTAGCACGGTCTTACCAT	ATATGAGGCGAGCAACTTGG
unigene15470	CCGGGGAAAACACCTAAAAT	TGCTCCAGGCTCTCTTTCTC
unigene101515	AGCTGGAGGAAGTACGGTCA	GTACACGACGTCGAAGAGCA
unigene32341	TGCAGGTATTGATGCTGCTC	GATCATGCGATGCTCTCTCA
unigene77434	TCTCCGAGGCATCAATCTTT	GCATGGTAGTCCCACAGGTC
unigene54524	TTTTGGACGGAGGGAGTACA	CCAGAGTCCCGAACAAGAGT
unigene81651	GCCACAGCGTGACAAGTACA	GATTCCACCAGCGACAACAA
unigene20153	GCCTCATGAACAGCTCCTCT	GAAACCCCATGACCTCAAGA
unigene667	CCACAGTCACCCAAACCTTC	TGTAGCAGTCCAGGTTGTCG
Unigene54218	GGGTTTGAGGGTTTGTGTTG	CCTGCATTCATGAACACGTC
Unigene133728	TGTCGTCCGAGTTGAATGAA	CTCCATCCTCGAGCTGCTAC
Unigene1969	CGTGGTGGACATAGACATGG	ACGTCGTCAGTGCCATCATA
Unigene19810	TCGTGGACATGAACCTCTGA	CGTGCCTAAAATGGCAAAAT
Unigene41043	GCACGCAAGTGTCATTCCTA	CGATGATTAATGAAGGGCAAT
Unigene57494	CGTGCCTATCCATGAGTGAG	GGCAGCCGTAGCTTAGTTTTT
Unigene61446	TTTGCACCCTCCTAGTGCTC	CATGGCCACCGTAATCTTCT
Unigene76429	CCTTGTGGCTTTTACCGTGT	AGCTGGCGCCACACATATAG
Unigene101216	GGAATGATACCCAACCATGC	CGCATCTTATCAGCGTCGTA
Unigene105290	CGCCGAGAATTAATCAGCAT	AAAATGATGGACGCATGACA
Unigene161070	TGAGAAGGGGATTGAGATGG	CTTGAAACCCGGAGAATCAG
Unigene17832	AGCGGCGTTAGGAGTCTAGC	TGCTCGATCAGCGAATCTTA
Unigene93802	TATTTCAGCAGCCCTATCAC	TGATTGATCCTGGTAGCATT

Table S4. ***In silico*** **mapping of genes with undetected expression in NAUH117 compared with that in Wangshuibai.**

**Unigenes**	**Chromosomes**	**Deletion bins on 3BS**
Unigene133056	**3BS** 3DS 4DL	**3BS FL0.78-1**
Unigene4383	3AS **3BS** 4DS	**3BS FL0.78-1**
Unigene18392	3AS **3BS** 3DS	**3BS FL0.78-1**
Unigene15240	3AS **3BS** 3DS	**3BS FL0.78-1**
Unigene125420	3AS **3BS** 3DS	**3BS FL0.78-1**
Unigene156462	3AS **3BS** 3DS	**3BS FL0.78-1**
Unigene15086	3AS **3BS**	**3BS FL0.78-1**
Unigene96231	3AS **3BS** 3DS	**3BS FL0.57-0.78**
Unigene25114	1AS 1DS **3BS**	**3BS FL0.33-0.57**
Unigene15248	3AL 3BL 3DL	-
Unigene161794	1BL 1DL	-
Unigene36081	1AL 2DL 3AL 3DL 5DS	-

Table S5. Twelve unigenes were detected to be up-regulated in Wangshuibai while were not expressed in NAUH117 based on Digital Gene expression assay and 8 out of them were confirmed by qRT-PCR.

Gene IDs	Gene annotations	Induced or not during *Fg* infection in Wangshuibai by qRT-PCR assay
Unigene93802	Receptor-like kinase	Induced
Unigene17832	Lipid transfer protein	Induced
Unigene41043	NADP-dependent oxidoreductase	Induced
Unigene61446	Glycine-rich protein	Induced
Unigene133728	ARK protein	Induced
Unigene57494	CBL-interacting protein kinase	Induced
Unigene1969	No similarity in nr database	Induced
Unigene161070	No similarity in nr database	Induced
Unigene101216	late embryogenesis abundant protein	Not Induced
Unigene76429	protein kinase	Not Induced
Unigene19810	Endo-1,4-beta-xylanase	Not Induced
Unigene105290	No similarity in nr database	Not Induced
