# Supplementary material for: Transcriptome-based discovery of pathways and genes related to resistance against Fusarium head blight in wheat landrace Wangshuibai
Source: BMC Genomics. 2013 Mar 21;14:197. doi: 10.1186/1471-2164-14-197 (PMC3616903; doi:10.1186/1471-2164-14-197)

Figure S1. Length distribution of unigenes.

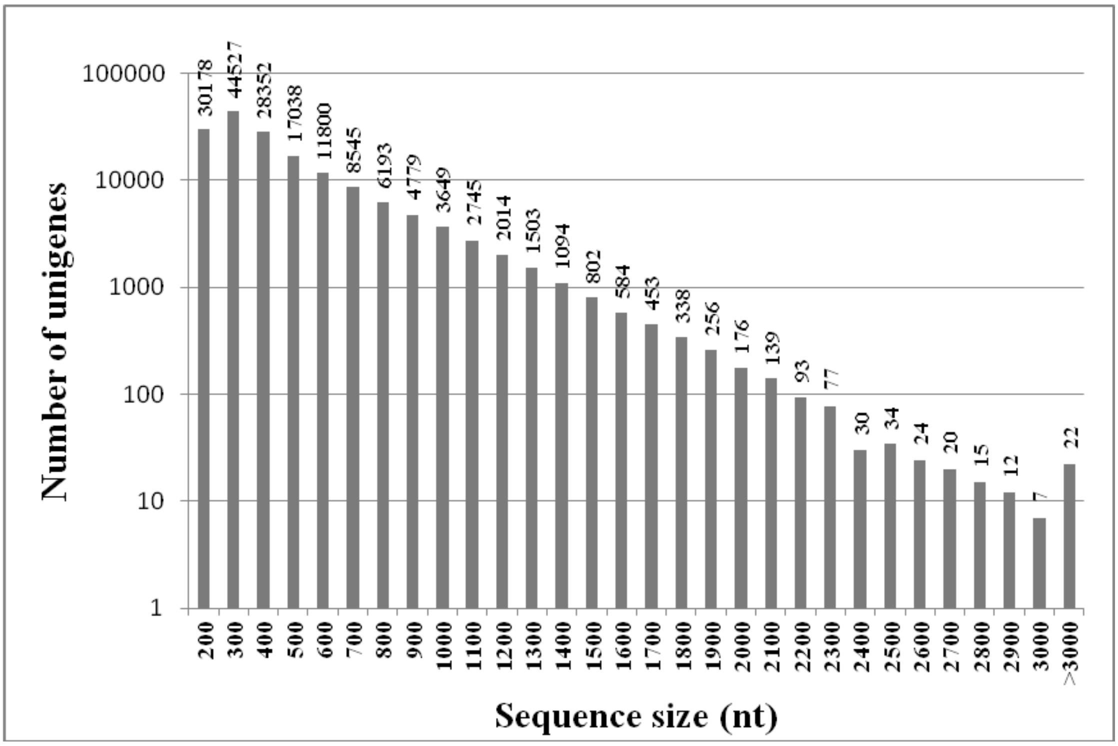

Figure S2. The length (A) and species (B) distribution of unigenes and the proportion of unigenes with matches in nr and Swiss port databases.

A

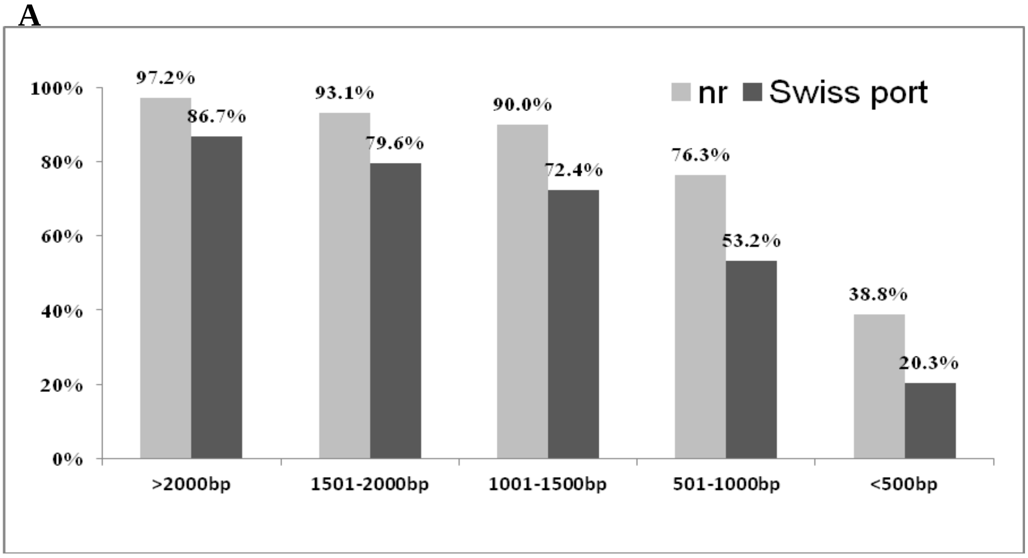

B

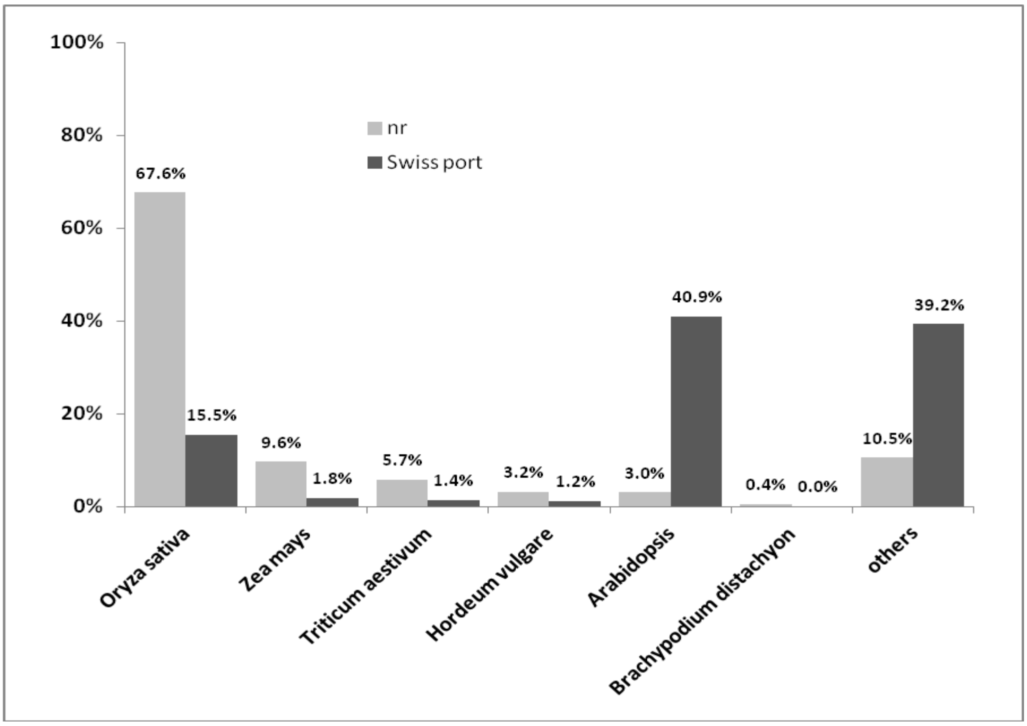

Figure S3. Saturation analysis of DGE sequencing. When the amount of tags reached 2M or higher, the increase of number of detected genes almost ceased for each library (indicated by the arrow).

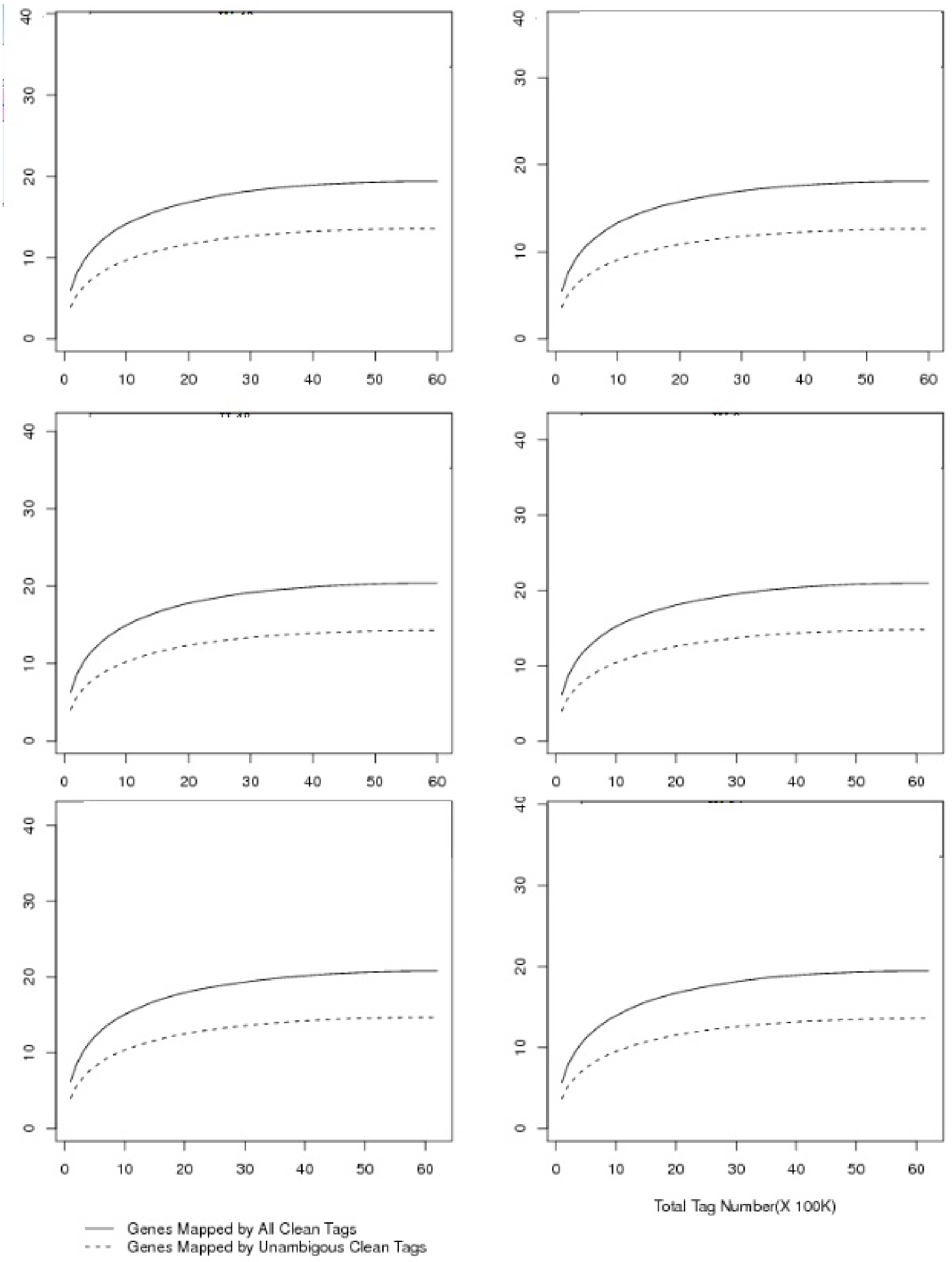

Supplement: Additional file 2: Figure S1 — Length distribution of unigenes. Figure S2. The length (A) and species (B) distribution of unigenes and the proportion of unigenes with matches in nr and Swiss port databases. Figure S3. Saturation analysis of DGE sequencing. When the amount of tags reached 2M or higher, the increase of number of detected genes almost ceased for each library (indicated by the arrow). [file 1471-2164-14-197-S2.pdf]
